# 

**Published:** 1893-08

**Authors:** 


### CONTENTS.

ORIGINAL COMMUNICATIONS—
CHOLERA. By Mr. Ernest Hart, London .... 321
ANTE AND RETRO-POSITIONS OF THE UTERUS—THEIR PATHOLOGY, SYMPTOMATOLOGY AND TREATMENT. By W. W. Stewart, M.D., Columbus, Ga .... 327
CLINICAL REPORT OF A CASE OF HEPATIC ABSCESS. By A. J. Bloch, M. D., New Orleans, La .... 337
WHAT THE GENERAL PRACTITIONER SHOULD KNOW ABOUT DISEASES OF THE EYE. By Frank Trester Smith, A.M., M.D., Chattanooga, Tenn .... 340
SOCIETY REPORTS—
ORLEANS PARISH MEDICAL SOCIETY .... 343
CORRESPONDENCE—
NEW YORK LETTER .... 350
CASE FOR DIAGNOSIS AND TREATMENT .... 354
EDITORIAL—
MEDICAL JOURNALISM .... 356
THE UNITED CONFEDERATE VETERANS .... 358
COMMENTS FROM THE WEST .... 359
SELECTIONS AND ABSTRACTS—
EYE, EAR, NOSE AND THROAT .... 361
OBSTETRICS AND GYNECOLOGY .... 364
SKIN DISEASES AND SYPHILIS .... 369
SURGERY .... 372
GENERAL MEDICINE AND THERAPEUTICS .... 376
MEDICAL ITEMS .... 379
BOOK REVIEWS .... 381
ADVERTISERS' INDEX TO ADVERTISEMENTS .... xii
CLASSIFIED INDEX TO ADVERTISEMENTS .... xlvii
ITEMS OF INTEREST .... xi
PREMIUM OFFERS .... xxxv

